# Protein craving links larval signals to food provisioning in honey bees

**DOI:** 10.1126/sciadv.aec3855

**Published:** 2026-05-13

**Authors:** Zhenfang Li, Yashuai Wu, Jiaming Liu, Chengfeng Yang, Shuai Wang, Min Huang, Shiqi Luo, Xin Zhou

**Affiliations:** State Key Laboratory of Agricultural and Forestry Biosecurity, MOA Key Lab of Pest Monitoring and Green Management, College of Plant Protection, China Agricultural University, Beijing 100193, China.

## Abstract

The provisioning of royal jelly for developing larvae by nurse bees is fundamental to social interaction in honey bee colonies. While royal jelly production is regulated by collective larval demand, it remains unclear how colony-level needs are translated into individual worker behavior. Here, we show that the larval pheromone E-β-ocimene (EBO), a volatile compound also used by pollinators as a floral food cue, elicits an intrinsic craving for protein in nurse bees that drives increased pollen consumption. Through in vitro and in vivo experiments, we demonstrate that this response is mediated by the leucokinin (*Lk*) and leucokinin receptor (*Lkr*) system, acting through the PKA-CREB-IRS signaling pathway to modulate the expression of the insulin receptor substrate gene (*Irs*). Elevated pollen intake then promotes the enlargement of the hypopharyngeal glands and enhanced production of major royal jelly proteins. Our findings uncover a molecular mechanism linking larval signaling to worker nutrition, highlighting how social bonds between honey bee larvae and nurses are rooted in ancestral pathways of protein hunger that predate eusociality.

## Teaser

Larval pheromone activates the *Lk*/*Lkr* pathway to couple nurse bee protein appetite with royal jelly production.

## INTRODUCTION

The division of labor and altruistic brood care form the foundation of honey bee sociality. Worker bees contribute to colony success through tasks such as nursing and foraging, which support brood development. Of the two principal food sources, pollen is the main source of protein required for larval growth (*1*, *2*). Because direct pollen consumption by larvae is negligible, larvae rely on nurse bees to ingest and metabolize pollen into protein-rich brood food (*3*–*6*). Thus, nurse bees serve as the colony’s primary consumers of pollen (*4*, *7*–*10*). Pollen-derived nutrients are converted into royal jelly components, which are synthesized and secreted by the hypopharyngeal glands (HPGs) (*11*, *12*). In this way, HPGs provide the proteins necessary for larval growth and colony development (*3*, *5*, *12*). The stimulatory effect of pollen on HPG development is conserved across social bees, including *Apis mellifera* (*12*, *13*), *Apis cerana* (*14*), and the stingless bee *Tetragonula pagdeni* (*15*). Moreover, the size of HPG development in nurse bees correlates strongly with larval survival and developmental capacity (*16*), making it a key indicator of larval food quality (*17*).

Larvae and nurses interact closely to balance brood nutritional needs with worker behavior. Colony-level protein demands are met through behavioral adjustments of individual workers (*18*, *19*), mediated by nurse inspections of brood cells (*20*) and brood pheromones (*19*, *21*, *22*). Among these mechanisms, pheromones provide particularly effective signals of collective larval nutritional demand. Honey bee brood produce two major pheromones, E-β-ocimene (EBO) and brood ester pheromone (*23*, *24*), with EBO being the most abundant (*25*). EBO is synthesized primarily by second- and third-instar larvae (*24*) and is released at elevated levels when larvae are hungry (*19*). Because EBO accumulation scales with larval numbers, its concentration modulates worker behaviors, particularly pollen foraging and brood care (*1*, *19*, *26*).

However, the mechanistic link between elevated larval EBO and increased food provisioning by nurses is still poorly understood. In particular, it remains unclear how larval signals are translated into molecular and behavioral responses in workers. Given that EBO is also naturally released by flowers to attract pollinators (*27*), we hypothesize that intensified larval EBO increases protein cravings in nurse bees. This response would increase pollen consumption and promote HPGs to produce royal jelly. Previous studies suggest roles for neuromodulators and insulin signaling in the regulation of feeding behavior (*28*), but their integration with larval signaling remains unresolved. In our recent work on *A. cerana*, we identified the leucokinin receptor (*Lkr*) as a key regulator of worker feeding motivation (*29*). We therefore propose that larval EBO acts through the leucokinin and leucokinin receptor (*Lk*/*Lkr*) system and its downstream signaling components to couple brood nutritional demand with nurse provisioning behavior.

Here, we demonstrate that honey bee larvae exploit ancestral neuropeptide and nutrient-sensing mechanisms to convey amino acid demand to worker bees. This signal stimulates intrinsic protein hunger in nurses, thereby driving pollen consumption and facilitating HPG enlargement, which enhances the synthesis and secretion of royal jelly. At the molecular level, we identify the *Lk*/*Lkr* system as the central hub that transduces larval EBO into worker responses by activating the insulin receptor substrate gene (*Irs*) pathway via a signaling cascade involving protein kinase A (PKA) and cAMP-response element binding protein (CREB). Our results reveal that collective larval demand directs individual worker behavior, offering insights into the molecular basis of honey bee sociality.

## RESULTS

### EBO enhances pollen consumption in Asian honey bees by activating *Lk*/*Lkr* signaling pathway

**Fig. 1.**
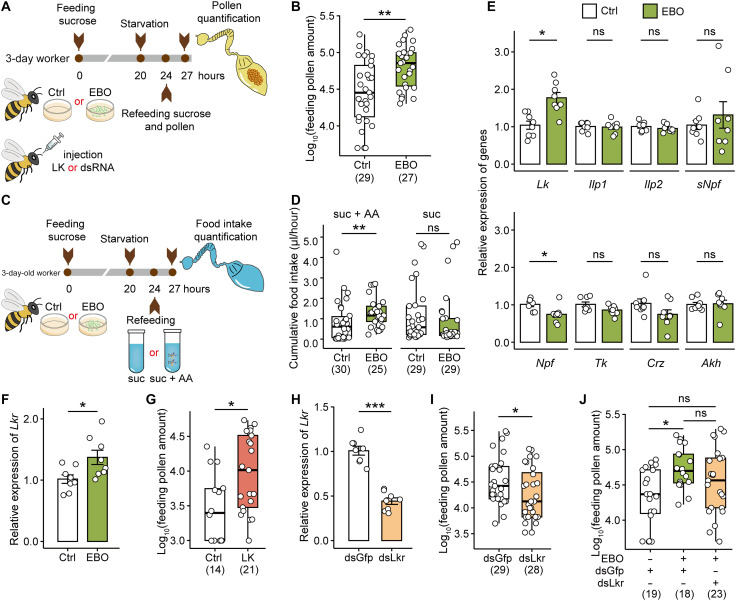
EBO promotes pollen and amino acid consumptions in Asian honey bees, with *Lk*/*Lkr* involved in downstream regulation. (**A**) Schematic illustration of bee treatment and pollen quantification. (**B**) EBO treatment increased pollen consumption. (**C**) Schematic illustration of measuring EBO impacts on amino acid consumption. (**D**) EBO treatment promoted bee intake of the amino acid–added sucrose solution, but not pure sucrose. (**E**) Expression level of the neuropeptide and peptide hormone genes in bee brains after EBO treatment. *Ilp1*, insulin-like peptide 1; *Ilp2*, insulin-like peptide 2; *sNpf*, short neuropeptide F; *Npf*, neuropeptide F; *Tk*, tachykinin; *Crz*, corazonin; *Akh*, adipokinetic hormone. (**F**) *Lkr* expression in bee brains increased after EBO treatment. (**G**) LK injection increased pollen intake. (**H**) Expression of *Lkr* in bee brains 24 hours after injection of *Lkr* dsRNA. Bees injected with GFP dsRNA were set as the control. (**I**) *Lkr* knockdown decreased pollen consumption. (**J**) Knockdown of *Lkr* reduced the promoting effect of EBO on pollen consumption. Each data point in (B), (D), (G), (I), and (J) represents the gut of one bee. Numbers in parentheses denote sample sizes. Each point in (E), (F), and (H) represents the brain of one bee. *n* = 8 biological replicates per group. Data are represented as means ± SEM. Statistical analyses in (B) and (D) to (I) were performed using a *t* test or Mann-Whitney test. Analysis in (J) was performed using the Kruskal-Wallis test with Dunn’s post hoc test. ns, not significant. *P < 0.05, **P < 0.01, and ***P < 0.001. suc, sucrose; AA, amino acid.

We demonstrated that EBO, a larval hunger signal, significantly increased pollen consumption in 4-day-old adult Asian honey bee *A. cerana* (Fig. 1, A and B). Because pollen is the main protein source for producing royal jelly, which nurse bees feed to larvae, we speculate that EBO-enhanced pollen consumption reflects an increased amino acid demand in workers. As expected, EBO exposure significantly elevated food consumption in adult bees only when provided with sucrose solution supplemented with amino acids, but not with a pure sucrose diet (Fig. 1, C and D).

Given the close association between neuropeptides and hormonal signaling in insect feeding regulation (*28*), we analyzed the expression of relevant genes. The reverse transcription quantitative polymerase chain reaction (RT-qPCR) results revealed that EBO specifically up-regulated *Lk* expression but down-regulated neuropeptide F (*Npf*) in the brains of worker bees among the genes examined (Fig. 1E). In addition, EBO also significantly increased the expression of *Lkr* (Fig. 1F). The Asian honey bee has a single LK gene producing multiple mature forms (*30*). However, it was unclear which mature peptide serves as the ligand of LKR. We synthesized one predicted mature form of LK and confirmed its binding to LKR recombinant proteins on the cell membrane (fig. S1, A to C). The injection of activated LK analogs in the brain increased pollen consumption in bees (Fig. 1G). Conversely, *Lkr* knockdown (Fig. 1H) significantly decreased pollen consumption in worker bees (Fig. 1I). Further experiments combining EBO treatment with *Lkr* RNA interference (RNAi) showed that *Lkr* knockdown abolished the EBO-induced increase in pollen feeding in adult bees, such that pollen consumption in the EBO + dsLkr group was comparable to that of the control group (Fig. 1J). These findings show that LK/LKR is involved in the regulation of EBO-induced pollen feeding behavior in honey bees.

### LK/LKR stimulates cellular ERK1/2 phosphorylation, Ca^2+^_,_ and cAMP concentration

To elucidate how LK modulates feeding behavior through its receptor, we investigated how LK and LKR transduces intracellular signals. LKR is a typical G protein–coupled receptor (GPCR). Upon activation by its ligand (in this case, LK), GPCRs primarily initiate multiple second-messenger pathways via downstream Gα protein subunits (such as Gαq and Gαs). This cascade leads to a series of cellular responses, such as protein phosphorylation [e.g., extracellular signal–regulated kinase1/2 (ERK1/2)], Ca^2+^ mobilization, and changes in cyclic adenosine monophosphate (cAMP) levels, thereby converting extracellular signals into intracellular responses (*31*). However, the specific Gα proteins involved in honeybee LKR signaling, along with their respective downstream effectors that ultimately regulate feeding, remain uncharacterized and require experimental validation in this system.

**Fig. 2.**
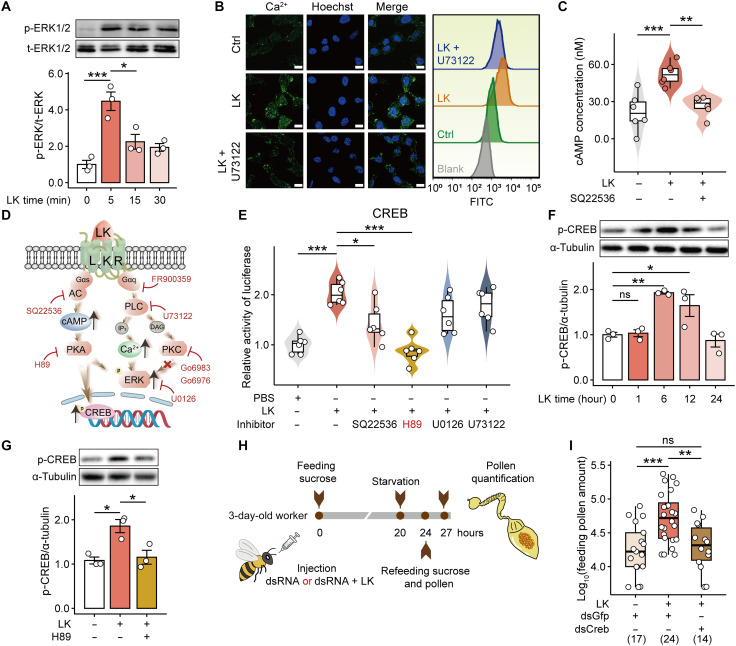
The binding of LK to LKR transmits extracellular signals into cells and regulates pollen consumption by activating the CREB transcription factor. (**A**) HEK293T cells expressing LKR showed an increase in ERK1/2 phosphorylation after LK treatment. (**B**) Ca^2+^ levels in HEK293T cells increased after LK-LKR binding, but this increase was inhibited by the PLC inhibitor U73122. The fluorescence intensity of the Ca^2+^ probe was measured using confocal microscopy and flow cytometry. (**C**) LK treatment significantly increased cAMP levels in HEK293T cells, while the AC inhibitor SQ22536 decreased cAMP levels. (**D**) Schematic illustration of the signaling pathway of LK and LKR in HEK293T cells. (**E**) Downstream CREB transcription factor is activated by LK and LKR. The inhibitors SQ22536 and H89 significantly inhibited CREB activity. HEK293T cells transfected with pCMV-N-FLAG-LKR and pCREB-TA-Luc plasmids were treated with LK and inhibitors, and the activity of firefly luciferase in the cells was measured. (**F**) LK increased the phosphorylation of CREB in honey bee heads, showing a time-dependent pattern. (**G**) H89 attenuated the activating effect of LK on CREB phosphorylation after 6 hours of LK treatment. (**H**) Schematic illustration of pollen consumption measurement after cotreatment with LK and *Creb1* knockdown. (**I**) LK supplementation increased pollen intake in bees, but this effect was reduced after *Creb1* knockdown. Each point in (A), (F), and (G) represents one biological replicate (*n* = 3). Data are represented as means ± SEM. Each point in (I) represents the gut of one bee. Numbers in parentheses denote sample sizes. Data in (A), (C), (E), (F), (G), and (I) were analyzed using ANOVA with the Tukey post hoc test. *P < 0.05, **P < 0.01, and ***P < 0.001. FITC, fluorescein isothiocyanate.

We found that LK treatment rapidly induced the phosphorylation of ERK1/2, peaking at 5 min (Fig. 2A). To dissect the upstream pathways, we used a series of specific inhibitors of the functional proteins. The Gαq protein inhibitor FR900359, phospholipase C (PLC) inhibitor U73122, adenylate cyclase (AC) inhibitor SQ22536, cAMP-dependent PKA inhibitor H89, and ERK1/2 inhibitor U0126 all blocked LK-induced ERK1/2 phosphorylation (fig. S2, A and B). In contrast, the protein kinase C (PKC) inhibitors Go6983 and Go6976 had no such effect (fig. S2B). This pharmacological profile suggests that LK/LKR activates ERK1/2 through two parallel pathways, Gαs → AC → PKA and the Gαq → PLC → Ca^2+^, thereby linking extracellular cues to intracellular responses.

The above inferences were confirmed at the level of second messengers. First, LK/LKR triggered the release of Ca^2+^ from the endoplasmic reticulum to the cytoplasm, which was inhibited by the PLC blocker U73122 (Fig. 2B). LK/LKR also increased intracellular cAMP levels, which were blocked by the AC inhibitor SQ22536 (Fig. 2C). Together, these results reveal a clear signaling transduction pathway for LK/LKR: LK binding and activation of its GPCR (LKR) activates both Gαq and Gαs proteins. Gαq elevates intracellular Ca^2+^ via PLC, while Gαs drives cAMP production and PKA activation via AC. The Ca^2+^ signal and the PKA signal cooperatively promote the phosphorylation of ERK1/2 (Fig. 2D), thereby establishing the link between extracellular cues and intracellular responses.

### LK/LKR signaling activates CREB

We examined changes in the three common downstream transcription factors, CREB, ETS transcription factor ELK1 (Elk1), and nuclear factor of activated T cells (NFAT) (*32*), after LK/LKR signal transduction into the cells. LK/LKR signaling activated CREB, Elk1, and NFAT, with CREB showing the strongest activation (Fig. 2E and fig. S3). In the signaling cascade leading to CREB activation, the activation of AC led to the production of cAMP, which in turn activated PKA through binding to its regulatory subunits. Activated PKA phosphorylated CREB at the conserved serine-133 (Ser^133^) site, enabling CREB to regulate the transcription of downstream target genes (*33*). The AC inhibitor SQ22536 and the PKA inhibitor H89 attenuated LK-induced CREB activation (Fig. 2E). The ERK inhibitor U0126 and the PLC inhibitor U73122 did not affect CREB activation, thus confirming the LK/LKR → AC → PKA → CREB pathway (Fig. 2D).

The PKA inhibitor H89 reduced CREB activity in vitro (Fig. 2E). We compared the two CREB proteins (LOC108001656 and LOC107995287) in the Asian honey bee genome and confirmed that only LOC108001656 (named CREB1 hereinafter) contains the conserved Ser^133^ sequence (fig. S4A), which is the phosphorylation site for PKA (*33*). In vivo, the LK analog induced CREB phosphorylation in the honey bee brain at 6 and 12 hours postinjection (Fig. 2F). The addition of the PKA inhibitor H89 reduced CREB phosphorylation (Fig. 2G), confirming the role of PKA in LK/LKR signaling.

To investigate the role of CREB in LK/LKR-regulated pollen feeding, we knocked down *Creb1* expression in honey bees using RNAi (fig. S4B). The pollen consumption of honey bees cotreated with LK and *Creb1* double-stranded RNA (dsRNA) was significantly lower than that of bees cotreated with LK and green fluorescent protein (GFP) dsRNA (*P* = 0.004; Fig. 2, H and I), but not significantly different from those treated with GFP dsRNA (Fig. 2I). These results indicate that CREB is a key downstream transcription factor, through which LK/LKR promotes pollen feeding behavior in the Asian honey bees.

### IRS is a direct transcriptional target of the CREB transcription factor

Subsequently, we sequenced transcriptomes of honey bee heads from three groups: LK treated, LK and H89 treated, and a control group. This allowed us to examine CREB-regulated genes in response to LK/LKR signaling (fig. S4, C to E), as CREB is activated via PKA-mediated phosphorylation, and H89 specifically blocks this pathway. LK activated CREB for at least 12 hours (Fig. 2F). Honey bees were then subjected to the treatments for 24 hours before examination to allow sufficient transcription of downstream target genes. Compared with the control group, LK treatment resulted in differential expression of 1790 genes, including 1164 up-regulated and 626 down-regulated genes (table S1). We focused on the LK-induced up-regulated genes and found that most of them (965 of 1164) were no longer up-regulated when the PKA inhibitor H89 was added (table S2), indicating that the inhibition of downstream CREB activity largely blocks LK-dependent transcriptional activation of these genes.

**Fig. 3.**
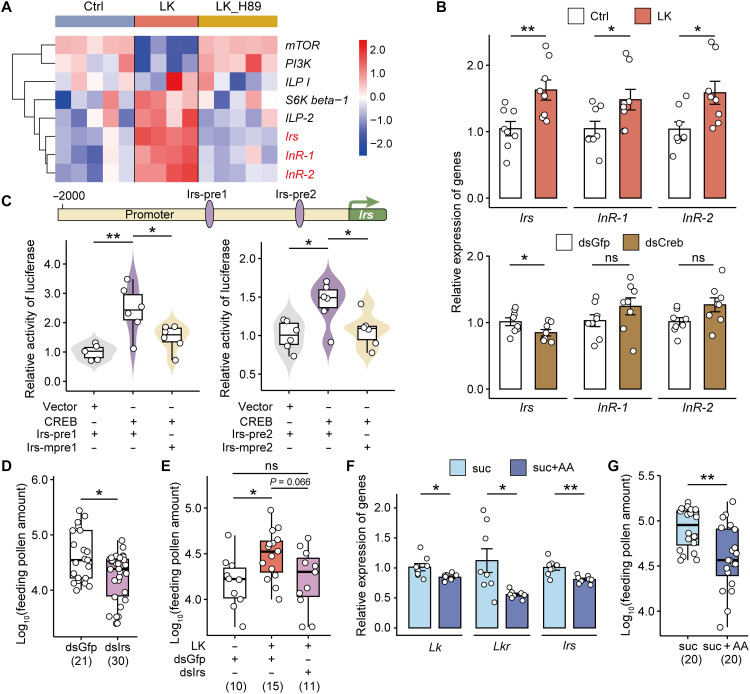
CREB activates *Irs* transcription and pollen feeding in Asian honey bees. (**A**) Heatmap of nutrient-related genes that were differentially expressed in the heads of bees treated with LK and the PKA inhibitor H89. (**B**) Expression levels of *Irs*, *InR-1*, and *InR-2* in the bee brain 24 hours after treatment with LK supplementation or *Creb1* RNAi. (**C**) Dual-luciferase system confirmed that CREB activated the transcription of two *Irs* promoter sequences. Purple ellipses: predicted CREB binding sites on the *Irs* promoter. HEK293T cells were transfected with pGL4.10 plasmids carrying the *Irs* promoter binding site sequence (Irs-pre1 or Irs-pre2) or binding site mutant sequence (Irs-mpre1 or Irs-mpre2) and pCMV-N-FLAG plasmids carrying the CREB protein coding sequence, with empty pGL4.10 as the control group. (**D** and **E**) Knockdown of *Irs* reduced pollen intake (D) and partially weakened the promotion effect of LK on pollen consumption (E). (**F**) Amino acid consumption inhibited the expression levels of *Lk*, *Lkr*, and *Irs* in the bee brain. (**G**) Impacts of amino acid consumption on pollen intake. Suc: 30% (w/w) sucrose solution; suc + AA: 30% (w/w) sucrose solution containing amino acid mixture. All food contained brilliant blue dye. Each data point in (B) and (F) represents the brain from one bee. *n* = 8 biological replicates per group. Data are presented as means ± SEM. Each point in (D), (E), and (G) represents the gut of one bee. Numbers in parentheses denote sample sizes. Data in (B), (D), (F), and (G) were analyzed using a *t* test or Mann-Whitney test. Data in (C) and (E) were analyzed using ANOVA with the Tukey post hoc test. *P < 0.05 and **P < 0.01.

We next examined key genes within the target of rapamycin (TOR) and insulin signaling pathways, which are closely associated with nutrient intake (*28*, *34*, *35*). Insulin signaling was modulated by LK and H89 treatments. Specifically, several genes—including the insulin-like peptide 2 (*Ilp-2*, LOC108003129), insulin receptor substrate (*Irs*, LOC108004059), insulin receptor 1 (*InR-1*, LOC108000589), and insulin receptor 2 (*InR-2*, LOC108002859)—were up-regulated by LK but down-regulated when H89 was coapplied (Fig. 3A). The RT-qPCR analysis confirmed that all genes, except *Ilp-2*, were up-regulated following LK treatment (Fig. 3B and fig. S5A). However, only the expression of *Irs* was reduced following *Creb1* knockdown (Fig. 3B). Bioinformatic analysis predicted two potential CREB-binding sites in the promoter region of *Irs* (fig. S5B). The binding of these sites with CREB was further verified using dual-luciferase reporter assays (Fig. 3C), suggesting that *Irs* is a direct downstream target of CREB. Furthermore, RT-qPCR confirmed that *Irs* expression increased following EBO treatment (fig. S5C) and decreased after *Lkr* knockdown (fig. S5D), collectively strengthening its role as a CREB-regulated gene downstream of LK/LKR signaling.

Knockdown of *Irs* expression (fig. S5E) reduced pollen intake in honey bees (Fig. 3D) and marginally attenuated the LK-induced enhancement, such that pollen consumption in the LK + dsIrs group was comparable to that of the control group (Fig. 3E). Therefore, the EBO-LK/LKR-CREB-IRS signaling cascade regulates pollen consumption in Asian honey bees. Meanwhile, the supplementation of amino acids to the sucrose solution significantly decreased expression levels of *Lk*, *Lkr*, and *Irs* in worker bees (Fig. 3F), resulting in less pollen in the gut (Fig. 3G). By activating the LK/LKR-IRS signaling pathway that senses amino acid deficiency, EBO increases pollen and amino acid consumption in worker bees, which co-opt the intrinsic protein demand mechanism in adult honey bees.

### Pollen consumption promotes HPG development in adult worker bees

**Fig. 4.**
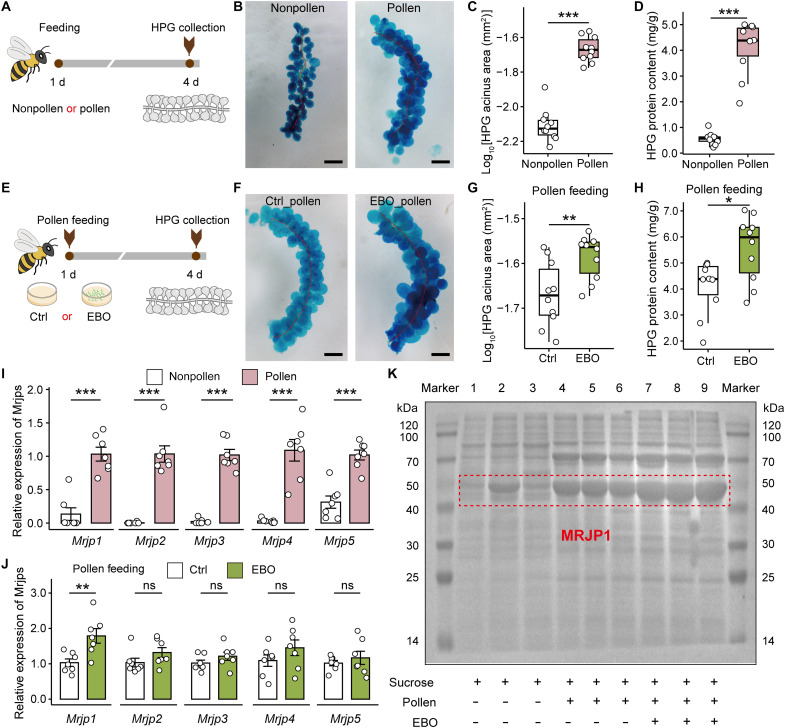
The development of HPGs in adult *A. cerana* workers is promoted by EBO-mediated pollen consumption. (**A**) Schematic illustration of the collection and measurement of HPG enlargement in adult worker bees provided with or without pollen. (**B**) HPG tissue stained with methylene blue from adult worker bees subjected to pollen-feeding or pollen-deprived treatments. Scale bars, 200 μm. (**C** and **D**) Pollen intake significantly increases HPG acinus size (C) and total protein content (D) in worker bees. (**E**) Schematic illustration of the collection and measurement of HPG enlargement in adult worker bees treated with EBO. (**F**) HPG tissue stained with methylene blue from adult worker bees treated with EBO, when the bees were maintained in a confined container and supplied with pollen diet. Scale bars, 200 μm. (**G** and **H**) EBO-induced pollen consumption enhances HPG acinus size (G) and total protein content (H) in worker bees. (**I**) Expression levels of *Mrjp1* to *Mrjp5* are significantly up-regulated in the HPGs of workers fed with pollen. (**J** and **K**) EBO-mediated pollen intake induces a marked increase in *Mrjp1* transcription (J) and protein expression (K). Each data point in (C) and (D) and (G) and (H) represents the HPG of one bee. *n* = 10 biological replicates per group. Each point in (I) and (J) represents the brain of one bee. Data are represented as means ± SEM. *n* = 7 biological replicates per group. Data in (C) and (D), (G) and (H), and (I) and (J) were analyzed using a *t* test or Mann-Whitney test. *P < 0.05, **P < 0.01, and ***P < 0.001.

**Fig. 5.**
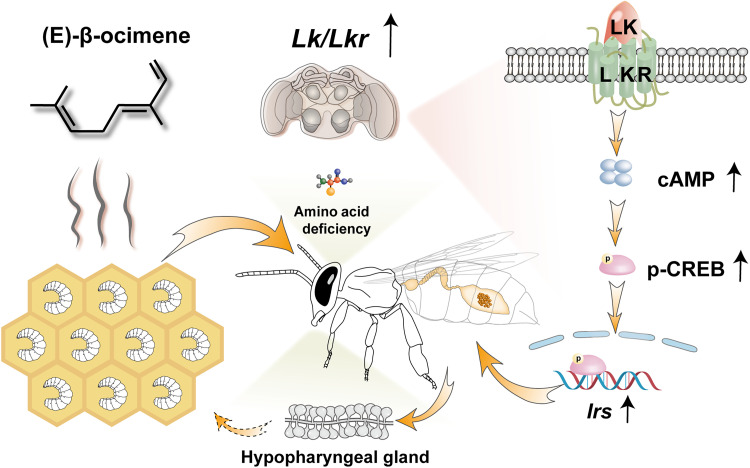
Model illustrating that EBO promotes HPG development in Asian honey bees by activating the LK/LKR signaling pathway, which increases pollen consumption. Young larvae in the hive release the hunger pheromone EBO, which conveys information for amino acid deficiency, leading to an increase in expression levels of *Lk* and *Lkr* in the brains of adult worker bees. These changes lead to an increase in intracellular cAMP levels, activating the transcriptional activity of CREB. Once activated, CREB promotes the transcription of *Irs*. The EBO-LK/LKR-CREB-IRS regulatory pathway prompts worker bees to increase pollen intake, thereby facilitating nutrient storage required for HPG development. Such a mechanism plays a crucial role in social interactions between honey bee larvae and adults.

Nurse bees are the primary consumers of pollen within a honey bee colony, and their key physiological role is to secrete royal jelly from the HPGs to nourish larvae and the queen (*4*, *7*). In our study, nurse bees provided with a pollen diet exhibited markedly enlarged HPG at 4 days of age compared to those deprived of pollen (Fig. 4, A and B). Pollen feeding resulted in a significant increase in both acinus size and total protein content of the HPGs (Fig. 4, C and D). Similarly, EBO-mediated pollen intake led to markedly enlarged HPGs (Fig. 4, E and F), as indicated by increased acinus size and protein content compared to the control group (Fig. 4, G and H). However, under pollen-deprived conditions, EBO treatment did not significantly enhance HPG development (fig. S6), suggesting that EBO alone is insufficient to activate HPG growth in the absence of dietary pollen. Subsequent RT-qPCR analysis revealed that the expression levels of *Mrjp1* to *Mrjp5*, the major royal jelly protein genes, were significantly up-regulated in the brains of pollen-fed bees (Fig. 4I). Furthermore, EBO-induced pollen consumption significantly up-regulated the transcription of *Mrjp1*, the most abundant component of major royal jelly proteins (Fig. 4J). This result was further supported by SDS–polyacrylamide gel electrophoresis (SDS-PAGE) analysis, which confirmed increased levels of MRJP1 protein (Fig. 4K). Collectively, our findings demonstrate that EBO functions as a larval hunger signal that stimulates pollen intake via LK/LKR-CREB-IRS axis in adult workers, thereby facilitating HPG development, an essential prerequisite for royal jelly production (Fig. 5).

## DISCUSSION

Our study shows that honey bee larvae signal their nutritional needs through the release of the volatile pheromone EBO, which elicits an intrinsic worker response to amino acid needs. Workers consume pollen and convert it into royal jelly in their HPGs, thereby sustaining brood development. From an evolutionary perspective, this provisioning behavior likely reflects a co-opted trait from solitary ancestors, where a single female carried out both reproduction and brood care.

EBO is also produced by flowering plants as a pollinator attractant (*27*), and only a few animals, including honey bees, butterflies, and mealworm beetles, synthesize β-ocimene compounds such as EBO and Z-β-ocimene (ZBO) (*19*, *24*, *36*–*40*). In insects, β-ocimene often serves as a chemical signal, attracting conspecifics, for example, in the butterfly *Tirumala limniace* (*36*) and the beetle *Alphitobius diaperinus* (*37*) or functioning as a male anti-aphrodisiac in *Heliconius melpomene* (*38*). By contrast, β-ocimene regulates food recruitment in bumblebees (ZBO) (*39*, *40*) and honey bees (EBO) (*1*, *19*, *26*, and this study). Social bees diverged from solitary ancestors 54 to 95 million years ago (*41*–*43*), lineages already adapted to pollen and nectar, where β-ocimene likely served as a floral cue (*27*, *44*). Solitary bee larvae develop on prestored provisions supplied by the ovipositing female (*45*), and no solitary bee species is known to produce β-ocimene. The acquisition of EBO production in ancestral social bees may thus have been pivotal for the evolution of eusociality, linking larval nutritional demand with worker provisioning.

We show that EBO enhances HPG development by favoring pollen intake over nectar, thereby increasing protein in worker bee diet, and protein-rich royal jelly secretion as food for larvae. These findings align with earlier reports that larval hunger signals or EBO elevate brood visitation by nurses (*18*, *19*), and complements studies indicating that EBO effects on HPGs vary with dosage, worker age, and interactions with other pheromones (*26*, *46*, *47*). Our results establish a “feeding-gland development” pathway as a mechanism coupling larval signals to worker provisioning.

At the molecular level, this interaction relies on conserved nutrient-sensing pathways. The *Lk*/*Lkr* signaling cascade transduces larval EBO into nurse worker responses by activating *Irs* expression through the PKA-CREB route. Using human embryonic kidney (HEK) 293T cells, we observed that LK/LKR activation triggers the cAMP-PKA-CREB signaling cascade. Although this heterologous system cannot fully mirror the complexity of signal transduction in the honey bee brain, it provides important support for the involvement of this conserved pathway. Future validation in honey bee cells will be informative once suitable cell lines become available. These results are further corroborated by in vivo experiments in honey bees, including CREB phosphorylation assays, RNAi, and behavioral analyses, collectively linking LK/LKR-dependent CREB activation to the regulation of pollen feeding behavior. However, whether the EBO-induced down-regulation of *Npf* contributes to feeding regulation remains unclear, and therefore, underlying molecular mechanisms warrant further investigation.

The *Lk/Lkr* pathway is widely implicated in feeding regulation across animals, from tardigrades to arthropods (*48*). In *Drosophila*, the ligand LK promotes protein intake (*49*), indicating a conserved role in amino acid regulation in at least holometabolous insects. Our recent analysis revealed that *Lkr* is under repeated selection in peripheral subspecies of Asian mainland *A. cerana* (*29*). Together, these findings suggest that pollen intake, tightly linked to brood production, has been a major target of selection during honey bee range expansion.

Downstream, *Irs* acts as a conserved insulin-related regulator (*50*). We show that LK activates the PKA-CREB-IRS cascade in the brain to promote pollen feeding. Prior work found that *Irs* is up-regulated in the brain and fat body of high pollen-hoarding strains and that RNAi knockdown of *Irs* in the fat body increases pollen load in foragers (*51*), indicating that *Irs* functions in a tissue- and stage-dependent manner.

Together, our study suggests that larval-worker communication evolved through the co-option of ancestral regulatory pathways. This created a physiological link between larvae and nurses comparable to interactions among organs within a “super-organism” (*52*, *53*). Studies of social evolution have often emphasized queen-worker differences or worker task allocation, often invoking the origin of novel genes and regulatory networks (*54*, *55*). Our results highlight a complementary principle: Conserved molecular pathways of solitary ancestors can be redeployed to mediate social communication, reinforcing the cohesion of insect societies.

Animals generally balance protein and carbohydrate intake to maintain health, and excess protein is often detrimental (*56*, *57*). In *Drosophila*, protein deprivation activates dopamine neurons, increasing protein intake while reducing sugar consumption (*58*). In *A. cerana*, sucrose sensitivity is modulated by *Lk/Lkr* (*29*), and the *Lk/Lkr* pathway suppresses sucrose intake (*59*). Here, we show that the same pathway also regulates protein intake. This suggests that the balance of protein and sugar intake may be coordinated through the *Lk/Lkr* hub, where signaling that promotes pollen feeding may simultaneously constrain nectar feeding.

In summary, EBO released by larvae conveys brood protein needs to workers, inducing pollen feeding and royal jelly synthesis. The *Lk/Lkr* system functions as the sensory hub that translates larval hunger signals into insulin pathway activity in workers. Because pollen intake underlies the pollination services provided by honey bees, these findings also have implications for the maintenance and improvement of pollination efficiency.

## MATERIALS AND METHODS

### Honey bee worker preparation

To obtain honey bee workers (*A. cerana*) for laboratory experiments, we purchased brood frames containing late-stage pupae from an apiary based in Changping District, Beijing, China. No specific permits were required for the collection or use of these bees, as they are managed for commercial and agricultural purposes and are not a protected or endangered species. We maintained the frame in a growth chamber at 34°C and 70% relative humidity to simulate conditions in managed colonies. After emergence, we transferred newly emerged bees to a plastic container and kept them under the same conditions. We supplied all worker bees with a 30% (w/w) sucrose solution for 3 days before experiments. As honey bees are invertebrates, specific ethical approval and an approval number were not required under the current national and institutional regulations in China. However, all effort were made to minimize the number of bees used and to ensure their welfare during the experiments.

### Sample collection and dissection

We anesthetized bees on ice and pinned them to a dissecting board using entomological pins. Under a stereomicroscope (SMZ745T, Nikon, Tokyo, Japan), we excised the head cuticle along the midline between the compound eyes using scissors. We dissected the HPGs surrounding the brain tissue and removed the pigmented tissues of both compound eyes to expose the intact brain. We then excised the brain and transferred it into prechilled phosphate-buffered saline (PBS), where we removed the surrounding connective tissues. Last, we immediately froze the intact brain tissues and HPGs in liquid nitrogen and stored them at −80°C until further use.

### Cell line

We obtained HEK293T cells (American Type Culture Collection, CRL-3216; RRID: CVCL_0063) from the American Type Culture Collection and maintained them at 37°C with 5% CO_2_ in complete Dulbecco’s modified Eagle’s medium (DMEM; Gibco, Thermo Fisher Scientific, MA, USA) supplemented with 10% fetal bovine serum (FBS; Zhejiang Tianhang Biotechnology, Huzhou, China).

### Treatment of honey bees with EBO and LK

We reared newly emerged Asian honey bees in a growth chamber for 3 days and then randomly assigned them to experimental groups receiving different combinations of EBO, LK injection, and RNAi of relevant genes. We determined sample sizes based on exploratory experiments and prior experience. We prepared EBO (W353901, Sigma-Aldrich, Merck, NJ, USA) by mixing it with paraffin oil (PX0047, Sigma-Aldrich, Merck, NJ, USA) at a ratio of 1:9 in a final volume of 1 ml. We placed 1 ml of the EBO mixture in the honey bee rearing container to enable gradual evaporation, whereas we treated control groups with 1 ml of paraffin oil alone.

We injected bees in the LK treatment group with 1 μl of LK solution (2 μg/μl) directly into the brain at the location between the three ocelli using a microsyringe (0.26 mm, Shanghai Bolige Industry & Trade Co. Ltd., Shanghai, China). To minimize brain tissue damage, we inserted the needle precisely without deep penetration. We prepared the LK solution by dissolving synthesized LK (FHWIOFNSWG-amide, SynPeptide, Shanghai, China) in enzyme-free sterile water [deoxyribonuclease (DNase)/ribonuclease (RNase)–free ddH_2_O]. We injected control bees with 1 μl of DNase/RNase-free ddH_2_O using the same procedure. We excluded individuals that died during the experiment from all analysis and did not include these samples in any statistical tests.

### Honey bee gene silencing

We designed primers for dsRNAs based on the coding sequence (CDS) of target genes and added a T7 promoter to the 5′ end of each primer (table S3). We used dsRNA targeting the GFP as a negative control. For PCR, we prepared a 50-μl reaction mixture containing 1 μl of cDNA synthesized from the brain of a single adult bee (as described in the “RNA extraction, reverse transcription, and RT-qPCR” section), 0.4 μM of each primer (table S3), and 2X Phanta Max Master Mix (Dye Plus) (P525, Vazyme, Nanjing, China). We conducted PCR according to the manufacturer’s protocol for the Phanta Max Master Mix (Dye Plus) kit. We then used the amplified PCR products of the target genes as templates for dsRNA synthesis. Last, we generated gene-specific dsRNAs using the T7 RiboMAX Express RNAi system (P1700, Promega, Madison, USA).

We injected 3-day-old adult honey bees directly into the head with specific amounts of dsRNA targeting *Lkr*, *Creb1*, or *Irs* gene (5 μg for *Lkr* RNAi, 2 μg for *Creb1* RNAi and 4 μg for *Irs* RNAi). We performed the injections following the same procedure described for LK treatment using GFP dsRNA at equivalent concentrations as a control. We then maintained the bees at 34°C and provided them with sucrose solution. After 24 hours of dsRNA treatment, we collected brains from honey bees in each group, stored them at −80°C, and used these samples to evaluate RNAi efficacy.

### Quantification of pollen consumption in gut

After EBO treatment, LK injection or gene RNAi, we placed honey bees in the rearing container and provided them with a 30% (w/w) sucrose solution for 20 hours. We then subjected the bees to a 4-hour starvation period in which they had access to water but no food. Following starvation, we supplied the bees with both a 30% (w/w) sucrose solution and a 61.5% (w/w) pollen diet (prepared by dissolving 8 g of pollen grains in 5 g of ddH_2_O) for 3 hours.

To quantify pollen consumption by workers, we dissected bee abdomens from each treatment group and transferred them into 1.5-ml centrifuge tubes. We thoroughly homogenized each abdomen in 500 μl of ddH_2_O, diluted the homogenate 10-fold, and used a subsample of the diluted mixture for microscopic pollen counting under a microscope. We placed a 1-μl drop of the pollen suspension onto a microscope slide for enumeration.

### Effects of amino acid supplementation on honey bee pollen intake and gene expression

To examine the effects of amino acid supplementation on honey bee pollen consumption, we prepared a 30% (w/w) sucrose solution and supplemented it with an amino acid mixture [minimum essential medium essential amino acid (MEM EAA) mix (50×), 11130051; MEM nonessential amino acid (100×), 11140050; and 200 mM glutamine, 25030081, Gibco, Thermo Fisher, MA, USA] to reach a final concentration of 2.5×. We provided this sucrose–amino acid mixture to newly emerged worker bees, whereas we supplied the control group with sucrose solution alone. After 20 hours of feeding on the amino acid mixture, we switched both the treatment and control groups to a water-only diet for 4 hours of starvation. We then offered the bees a sucrose solution together with a pollen mixture. After 3 hours of feeding, we quantified the amount of pollen consumed as described in the “Quantification of pollen consumption in gut” section.

After 24 hours of feeding on the amino acid mixture, we dissected the bees, collected their brains, and immediately frozen the samples for storage before RNA extraction and gene expression analyses to assess the effects of amino acid supplementation on gene expression.

### Effects of EBO on amino acid and sucrose intake in honey bees

To examine the effects of EBO on honey bee amino acid intake, we placed EBO-treated and control honey bees in the rearing container and provided them with a 30% (w/w) sucrose solution for 20 hours. We then subjected the bees to a 4-hour starvation period during which they had access to water but no food. After starvation, we offered the bees a sucrose solution containing a 2.5× amino acid mixture supplemented with brilliant blue (4 μg/μl; Erioglaucine disodium salt, 861146, Sigma-Aldrich, Merck, NJ, USA), which visually colored the diet blue. After 3 hours of feeding, we collected bee abdomens and placed them in 500 μl of ddH_2_O. We homogenized the tissues, centrifuged the homogenate, filtered the supernatant, and diluted it for analysis. We measured absorbance at 625 nm using a microplate reader (i3x, Molecular Devices, Danaher, WAS, USA). We generated a standard curve using brilliant blue solutions at concentrations of 4, 2, 1, 0.5, 0.25, 0.125, 0.0625, and 0 ng/μl and calculated amino acid intake from this curve to estimate sucrose solution consumption per bee per unit time. To examine the effects of EBO on honey bee sucrose intake, we provided EBO-treated and control bees with a sucrose solution containing brilliant blue and quantified sucrose consumption using the same procedure as for amino acid intake.

### Measurement of HPG acinus size and protein content

We maintained newly emerged 1-day-old worker bees in an incubator and provided them with pollen (as described above) and a 30% (w/w) sucrose solution. For the control group, we withheld pollen but supplied sucrose solution ad libitum. We reared both groups under sufficient food supply until day 4. Thereafter, we anesthetized bees on ice, dissected their HPGs, and collected the tissues as described in the “Sample collection and dissection” section.

To obtain clear visualization of the HPGs, we followed a previously described protocol with minor modifications (*13*). Briefly, we fixed freshly dissected intact HPGs in 4% paraformaldehyde solution (P1110, Solarbio, Beijing, China) for 5 min and then stained them with 0.1% methylene blue solution for 5 min. We removed excess stain by washing the tissues with PBS. We photographed the HPGs using an Image Analysis System 11 (Crisoptical, Beijing, China) at a resolution of 5440 × 3648 under a stereomicroscope (SMZ745T, Nikon, Tokyo, Japan). Under clear visual fields, we randomly selected 10 acini from each sample and measured their areas to calculate the mean acinus size. We analyzed 10 biological replicates per treatment group.

We extracted total proteins from HPGs using radioimmunoprecipitation assay (RIPA) buffer (R0010, Solarbio, Beijing, China) supplemented with protease inhibitor phenylmethylsulfonyl fluoride (PMSF) (R0010, Solarbio, Beijing, China). We quantified total protein content with a BCA protein assay kit (P0010S, Beyotime, Shanghai, China). We then prepared protein samples using the SDS-PAGE loading buffer (P0015, Beyotime, Shanghai, China) and loaded 30 μg of protein per lane onto a 12% SDS-PAGE gel (P0012A, Beyotime, Shanghai, China) for electrophoretic separation. After electrophoresis, we rinsed the gel three times (5 min each) with water to remove residual SDS that could contribute to background staining. We subsequently incubated the gel with 40 ml of SolarFast SDS-PAGE Reagent (G4540, Solarbio, Beijing, China) for 1 hour with gentle shaking, followed by extensive washing in water for 2 hours at room temperature until a clear background was achieved. Last, we imaged the gel using a Chemiluminescent Imaging System (C150, Azure, CA, USA).

### RNA extraction, reverse transcription, and RT-qPCR

We extracted total RNA from a single honey bee head or brain using RNA isolator Total RNA Extraction Reagent (R401-01, Vazyme, Nanjing, China). We added Dr. GenTLE Precipitation Carrier (9094, Takara, Shiga, Japan) to facilitate RNA precipitation. We dissolved the purified RNA in 15 μl of RNase-free water and used 2 μl of this RNA for cDNA synthesis with the PrimeScript RT reagent Kit with genomic DNA Eraser (RR047A, Takara, Shiga, Japan) in a 20-μl reaction volume. We then diluted the resulting cDNA fivefold for downstream analyses.

We performed each 10-μl RT-qPCR reaction with 1 μl of cDNA template, 0.2 μM of each primer (table S4), and 5 μl of Taq Pro Universal SYBR qPCR Master Mix (Q712, Vazyme, Nanjing, China). We conducted thermal cycling with an initial denaturation at 95°C for 3 min, followed by 40 cycles of denaturation at 95°C for 10 s and annealing/extension at 60°C for 30 s. To generate melting curves, we heated samples to 95°C for 15 s, cooled them to 60°C for 60 s, and then reheated them to 95°C for 15 s. We used the Asian honey bee *actin* gene, amplified with primers from a previously published study (*60*), as the reference gene. We calculated relative expression levels using the 2^–ΔΔCT^ method (*61*). We ran each sample in technical triplicate on the same plate.

### Plasmid construction

We constructed the pCMV-C-EGFP-LKR plasmid. We used full-length honey bee cDNA as a template and amplified the CDS of *Lkr* (LOC10799839) with the primers LKR-EGFP-F and LKR-EGFP-R (table S5). We performed the PCR in a 50-μl reaction containing 0.4 μM of each primer, cDNA synthesized from 20 ng of total RNA, and 25 μl of Phanta Max Master Mix (Dye Plus) kit (P525, Vazyme, Nanjing, China), following the manufacturer’s protocol. We then inserted the amplified CDS into the pCMV-C-EGFP plasmid (D2626, Beyotime, Shanghai, China) at the N terminus of enhanced GFP (EGFP) using homologous recombination (ClonExpress Ultra One Step Cloning Kit, C115, Vazyme, Nanjing, China) together with the restriction enzymes Hind III–HF (R3104V, NEB, MA, USA) and Pst I–HF (R3140V, NEB, MA, USA).

We constructed the pCMV-N-FLAG-LKR and pCMV-N-FLAG-CREB1 plasmids by amplifying the CDSs of *Lkr* (LOC10799839) and *Creb1* (LOC108001656) from full-length honey bee cDNA. We amplified *Lkr* using the primers LKR-FLAG-F and LKR-FLAG-R and *Creb1* using the primers CREB-FLAG-F and CREB-FLAG-R (table S5). We inserted the amplified CDSs into the pCMV-N-FLAG plasmid (D2722, Beyotime, Shanghai, China) at the C terminus of the FLAG tag using the restriction enzymes Hind III–HF (R3104V, NEB, MA, USA) and Eco RI–HF (R3101V, NEB, MA, USA).

We used the JASPAR transcription factor prediction database (https://jaspar.elixir.no) (*62*) to predict the CREB binding sites in the promoter region of the Asian honey bee *Irs* gene (LOC108004059). We constructed two reporter plasmids, Irspre1-pGL4.10 and Irspre2-pGL4.10, by amplifying the predicted binding sites together with their flanking regions using the primers Irspre1-pGL4.10-F/R and Irspre2-pGL4.10-F/R (table S5). We inserted these fragments into the pGL4.10 plasmid (9PIE665, Promega, Madison, USA) at the N terminus of the firefly luciferase gene using the restriction enzymes Hind III–HF (R3104V, NEB, MA, USA) and Eco RI–HF (R3101V, NEB, MA, USA). We generated plasmids carrying mutated binding sites using the Mut Express II Fast Mutagenesis Kit V2 (C214, Vazyme, Nanjing, China) and the primers listed in table S5.

### The subcellular localization of LKR and LK

We seeded HEK293T cells onto tissue-cultured glass coverslips in six-well plates and cultured them in complete DMEM medium (11965092, Gibco, Thermo Fisher Scientific, MA, USA) supplemented with 10% FBS (13011-8611, Zhejiang Tianhang Biotechnology, Huzhou, China) for 24 hours. We then transfected the cells with the pCMV-C-EGFP-LKR plasmid (2 μg per well) using Lipofectamine 3000 (L3000015, Thermo Fisher Scientific, MA, USA). After 36 hours, we treated the transfected cells with 2 μM LK peptide (TAMRA-FHWIOFNSWG-amide, synthesized by SynPeptide, Shanghai, China) for 10 min. We subsequently stained the cells with the nuclear dye Hoechst 33342 (C1025, Beyotime, Shanghai, China) 10 min and washed them with PBS to remove excess reagent. Last, we visualized cellular fluorescence signals using a laser confocal microscope (SP8, Leica, Danaher, WAS, USA).

### Western blotting

We cultured HEK293T cells in six-well plates and transfected them with 2 μg of pCMV-N-FLAG-LKR plasmid per well after 24 hours. Thirty-six hours posttransfection, we switched the cells to serum-reduced medium (Opti-MEM, 11058021, Gibco, Thermo Fisher Scientific, MA, USA) and maintained them for 4 hours to reduce basal ERK1/2 phosphorylation levels. We then treated the cells with 2 μM LK peptide (FHWIOFNSWG-amide, synthesized by SynPeptide) for 5, 15, or 30 min while leaving a control group untreated. In inhibitor experiments, we pretreated cells with the specified inhibitor (table S6) before LK treatment.

We prepared cell lysates to extract total proteins using RIPA buffer (R0010, Solarbio, Beijing, China) supplemented with protease inhibitor PMSF (R0010, Solarbio, Beijing, China) and protein phosphatase inhibitor (P1260, Solarbio, Beijing, China) to prevent dephosphorylation. We determined protein concentration using a BCA protein assay kit (P0010S, Beyotime, Shanghai, China) and prepared samples with SDS-PAGE loading buffer (P0015, Beyotime, Shanghai, China). We loaded 20 μg of protein per lane onto a 12% SDS-PAGE gel (P0012A, Beyotime, Shanghai, China) for electrophoretic separation.

We incubated membranes overnight at 4°C with primary antibodies: rabbit monoclonal anti–phospho-ERK1/2 (Thr^202^/Tyr^204^) antibody (1:1000; 4370, RRID: AB_2315112, Cell Signaling Technology, MA, USA) and rabbit monoclonal anti-ERK1/2 antibody (1:1000; 4695, RRID: AB_390779, Cell Signaling Technology, MA, USA). After incubation with goat anti-rabbit immunoglobulin G (IgG) secondary antibody (1:2000; SA00001-2, RRID: AB_2722564, Proteintech, IL, USA) for 1 hour at room temperature, we exposed membranes to enhanced chemiluminescence substrate (P0018M, Beyotime, Shanghai, China) and imaged them using a Chemiluminescent Imaging System (C600, Azure, CA, USA). We quantified band intensity using the ImageJ v1.54d (National Institutes of Health, Bethesda, MD, USA) (*63*).

We reared worker bees for 3 days, immobilized them on ice, and injected 1 μl of LK peptide (2 μg/μl) directly into the brain. We collected heads at 1, 6, 12, and 24 hours postinjection; control bees received 1 μl of ddH_2_O. In H89 inhibition experiments, we first injected 1 μl of 100 μM H89 into bee brains, followed by LK injection, and collected heads after 6 hours. We extracted total protein using RIPA lysis buffer containing PMSF and protein phosphatase inhibitors and determined protein concentration using a BCA protein assay kit. We loaded 60 μg of protein per lane and incubated membranes overnight at 4°C with rabbit monoclonal anti–phospho-CREB (Ser^133^) antibody (1:500; 9198, RRID: AB_2561044, Cell Signaling Technology, MA, USA) and mouse monoclonal alpha tubulin antibody (1:2000 dilution; 66031-1-Ig, RRID: AB_11042766, Proteintech, CA, USA). We then incubated membranes with secondary antibodies (goat anti-rabbit IgG, 1:2000; SA00001-2, RRID: AB_2722564; goat anti-mouse IgG, 1:2000; SA00001-1, RRID: AB_2722565, Proteintech, IL, USA) for 1 hour at room temperature. We imaged the bands and quantified grayscale values using ImageJ v1.54d (National Institutes of Health, Bethesda, MD, USA) (*63*).

### RNA sequencing and expression analysis

We assessed RNA quality using an Agilent 2100 Bioanalyzer (Agilent Technologies, CA, USA) and RNase-free agarose gel electrophoresis. We prepared libraries and performed transcriptome sequencing on an Illumina NovaSeq 6000 platform (PE150) at Novogene (Tianjin, China). We sequenced transcriptomes from three independent groups: (i) bees injected with 2 μg of LK for 24 hours, (ii) bees coinjected with 2 μg of LK and 100 μM PKA inhibitor H89 for 24 hours, and (iii) bees injected with ddH_2_O as a control. Each group contained four to five biological replicates, with each replicate consisting of the head of a single honey bee.

We filtered raw reads with low quality (quality score < 20 in more than 10% of bases) using fastp (v0.20.1) with parameters -q 20 -u 10 (*64*). We evaluated sequencing quality for each sample using FastQC (v0.11.8) with default settings (*65*). We aligned clean reads to the *A. cerana* reference genome (AcerK_1.0) using HISAT2 (v2.1.0) with parameters -p 20 --dta-cufflinks --no-mixed --no-discordant (*66*). We assembled aligned reads from each sample using StringTie (v2.0.6) with default settings (*67*) and extracted gene-level read counts using the prepDE.py3 script from the StringTie package. We performed differential gene expression analysis using DESeq2 in R (*68*) and defined genes with an adjusted *P* value < 0.05 and |log_2_ fold change| ≥ 1 as differentially expressed genes (DEGs).

### Detection of intracellular Ca^2+^ levels

We prepared a 3 μM working solution of the Ca^2+^ fluorescent probe Fluo-4 AM (IF1500, Solarbio, Beijing, China) by mixing it with an equal volume of 20% (w/v) Pluronic F127 (P6790, Solarbio, Beijing, China) to enhance solubility and facilitate cellular loading. We added this mixture to HEK293T cells expressing LKR, ensuring that the cells were fully immersed in the Fluo-4 AM solution (300 μl per well for confocal culture dishes and 800 μl per well for six-well plates). We incubated the cells at 37°C for 30 min, then washed them with PBS, and replaced the medium with opti-MEM (11058021, Gibco, Thermo Fisher Scientific, MA, USA) for 20 min to allow intracellular esterases to cleave Fluo-4 AM into Fluo-4, which remained trapped inside the cells.

After removing the culture medium, we stained the cells with Hoechst 33342 (C1025, Beyotime, Shanghai, China) in the dark and washed them with PBS. We then exposed the cells to LK for 5 min and visualized changes fluorescence intensity using confocal microscopy. For quantitative analysis by flow cytometry (BD FACSCelesta, NJ, USA), we detached cells with trypsin (T1300, Solarbio, Beijing, China), washed them, and resuspended them in PBS. We kept the cells on ice in the dark, treated them with LK for 5 min, and analyzed 10^4^ cells per sample to quantify intracellular Ca^2+^ levels. In inhibitor treatment groups, we added inhibitors to the cells before Fluo-4 AM loading. We analyzed all flow cytometry data using FlowJo X software (v10.0.7).

### Detection of intracellular cAMP levels

We seeded HEK293T cells expressing LKR in 96-well culture plate (354651, Corning, NY, USA) at a density of 10^4^ cells per well and incubated them overnight. We then treated the cells with an induction buffer containing 2 μM LK for 5 min in opti-MEM supplemented with 500 μM IBMX (HY-12318, MCE, NJ, USA) and 100 μM Ro 20-1724 (HY-100927, MCE, NJ, USA) to inhibit phosphodiesterase and prevent cAMP degradation. For inhibitor experiments, we pretreated cells with 100 μM SQ22536 (table S6) for 30 min before LK treatment, whereas control cells received no LK treatment. We measured intracellular cAMP levels using a cAMP-Glo Assay kit (V1501, Promega, Madison, USA). We lysed cells at room temperature for 15 min, followed by incubation with the cAMP detection reagent for 20 min. We then added the luciferase-substrate mixture and incubated for 10 min. We measured chemiluminescence intensity using a microplate reader (i3x, Molecular Devices, Danaher, WAS, USA). We generated a standard curve using the cAMP standard supplied with the kit and calculated sample cAMP concentrations based on this standard curve.

### Luciferase reporter assay

To investigate how LK and LKR influence the activity of different transcription factors, we cotransfected HEK293T cells with the pCMV-N-FLAG-LKR plasmid, firefly luciferase reporter plasmids carrying binding elements for specific transcription factors (pCREB-TA-Luc, D4050; pNFAT-TA-Luc, D4218; pElk1-TA-Luc, D4064, Beyotime, Shanghai, China.), and a Renilla luciferase reference plasmid (pGL4.73, 9PIE691, Promega, Madison, USA) at a 3:3:1 ratio. After 36 hours of transfection, we treated the cells with the indicated inhibitors and LK. We then lysed cells and measured firefly luciferase and Renilla luciferase activities using the Dual Luciferase Reporter Assay Kit (DL101, Vazyme, Nanjing, China) in a microplate reader.

To investigate CREB-mediated activation of the *Irs* promoter, we transfected HEK293T cells with the pCMV-N-FLAG-CREB1 plasmid, firefly luciferase reporter plasmids containing wild-type or mutated CREB binding sites from the *Irs* promoter (Irspre1-pGL4.10, Irspre2-pGL4.10, mut-Irspre1-pGL4.10, and mut-Irspre2-pGL4.10; PGL4.10 from 9PIE665, Promega, Madison, USA), and the Renilla luciferase reference plasmid (pGL4.73, 9PIE691, Promega, Madison, USA) at a 3:3:1 ratio. Thirty-six hours after transfection, we quantified luciferase activity using the same Dual Luciferase assay described above.

### Quantification and statistical analysis

We performed statistical analyses and generated graphs in R studio (R version 4.4.2) (*69*). For comparison between two groups, we used *t* test when data met assumptions of normality and homogeneity of variance; otherwise, we applied the Mann-Whitney test. For comparisons among multiple groups, we conducted one-way analysis of variance (ANOVA), followed by Tukey’s post hoc test when parametric assumptions were satisfied. When assumptions of normality or homogeneity of variance were not met, we instead used the Kruskal-Wallis test followed by Dunn’s post hoc test for pairwise comparison. We considered all pairwise comparisons statistically significant at *P* < 0.05.

## References

[1] K. S. Traynor, Y. Le Conte, R. E. Page, Age matters: Pheromone profiles of larvae differentially influence foraging behaviour in the honeybee, *Apis mellifera*. Anim. Behav. 99, 1–8 (2015).25580017

[2] T. Schmickl, K. Crailsheim, Inner nest homeostasis in a changing environment with special emphasis on honey bee brood nursing and pollen supply. Apidologie 35, 249–263 (2004).

[3] R. Brodschneider, K. Crailsheim, Nutrition and health in honey bees. Apidologie 41, 278–294 (2010).

[4] G. A. Wright, S. W. Nicolson, S. Shafir, Nutritional physiology and ecology of honey bees. Annu. Rev. Entomol. 63, 327–344 (2018). 29029590 10.1146/annurev-ento-020117-043423

[5] R. E. Snodgrass, A. M. Roger, *Anatomy of the Honey Bee* (Cornell Univ. Press, 2018).

[6] M. L. Winston, *The Biology of the Honey Bee* (Harvard Univ. Press, 1987).

[7] K. Crailsheim, L. H. W. Schneider, N. Hrassnigg, G. Bühlmann, U. Brosch, R. Gmeinbauer, B. Schöffmann, Pollen consumption and utilization in worker honeybees (*Apis mellifera carnica*): Dependence on individual age and function. J. Insect Physiol. 38, 409–419 (1992).

[8] R. R. Sagili, T. Pankiw, Effects of protein-constrained brood food on honey bee (*Apis mellifera* L.) pollen foraging and colony growth. Behav. Ecol. Sociobiol. 61, 1471–1478 (2007).

[9] D. E. Grogan, J. H. Hunt, Age correlated changes in midgut protease activity of the honeybee, *Apis mellifera* (Hymenoptera: Apidae). Experientia 36, 1347–1348 (1980).

[10] B. Moritz, K. Crailsheim, Physiology of protein digestion in the midgut of the honeybee (*Apis mellifera* L.). J. Insect Physiol. 33, 923–931 (1987).

[11] V. Corby-Harris, C. A. D. Meador, L. A. Snyder, M. R. Schwan, P. Maes, B. M. Jones, A. Walton, K. E. Anderson, Transcriptional, translational, and physiological signatures of undernourished honey bees (*Apis mellifera*) suggest a role for hormonal factors in hypopharyngeal gland degradation. J. Insect Physiol. 85, 65–75 (2016). 26658137 10.1016/j.jinsphys.2015.11.016

[12] G. DeGrandi-Hoffman, Y. Chen, E. Huang, M. H. Huang, The effect of diet on protein concentration, hypopharyngeal gland development and virus load in worker honey bees (*Apis mellifera* L.). J. Insect Physiol. 56, 1184–1191 (2010). 20346950 10.1016/j.jinsphys.2010.03.017

[13] V. Corby-Harris, L. A. Snyder, Measuring hypopharyngeal gland acinus size in honey bee (*Apis mellifera*) workers. J. Vis. Exp. 139, e58261 (2018). 10.3791/58261 PMC6235179 30272666

[14] R. Liang, C. Liang, Y. Zhang, J. Huang, G. Ding, Influence of different diets on growth and development of eastern honey bee (*Apis cerana*). Insects 16, 383 (2025). 40332865 10.3390/insects16040383 PMC12028326

[15] L. Straub, T. Sittisorn, J. Butdee, W. Promsart, A. Rueangwong, D. Camenzind, J. Maitip, Age-dependent hypopharyngeal gland size and protein content of stingless bee workers, *Tetragonula pagdeni*. PLOS ONE 19, e0308950 (2024). 39150928 10.1371/journal.pone.0308950 PMC11329107

[16] C. Şurlea, G. Diniță, M. Maftei, I. Marin, C. G. Nicolae, Study on the viability of the young in the species *Apis mellifera* according to the secretory capacity of royal jelly. Sci. Papers Ser. D Anim. Sci. 2, 274–278 (2022).

[17] V. Ghasemi, Effect of different nutritional diets on hypopharyngeal glands growth in nurse honey bees. J. Entomol. Soc. Iran 41, 273–278 (2021).

[18] C. Heimken, P. Aumeier, W. H. Kirchner, Mechanisms of food provisioning of honeybee larvae by worker bees. J. Exp. Biol. 212, 1032–1035 (2009). 19282500 10.1242/jeb.022582

[19] X. J. He, X. C. Zhang, W. J. Jiang, A. B. Barron, J. H. Zhang, Z. J. Zeng, Starving honey bee (*Apis mellifera*) larvae signal pheromonally to worker bees. Sci. Rep. 6, 22359 (2016). 26924295 10.1038/srep22359 PMC4770327

[20] Z. Y. Huang, G. W. Otis, Inspection and feeding of larvae by worker honey bees (Hymenoptera: Apidae): Effect of starvation and food quantity. J. Insect Behav. 4, 305–317 (1991).

[21] Y. Le Conte, A. Mohammedi, G. E. Robinson, Primer effects of a brood pheromone on honeybee behavioural development. Proc. Biol. Sci. 268, 163–168 (2001). 11209886 10.1098/rspb.2000.1345 PMC1088586

[22] R. R. Sagili, T. Pankiw, B. N. Metz, Division of labor associated with brood rearing in the honey bee: How does it translate to colony fitness? PLOS ONE 6, e16785 (2011). 21347428 10.1371/journal.pone.0016785 PMC3035648

[23] Y. Le Conte, G. Arnold, J. Trouiller, C. Masson, B. Chappe, Identification of a brood pheromone in honeybees. Naturwissenschaften 77, 334–336 (1990).

[24] A. Maisonnasse, J. C. Lenoir, G. Costagliola, D. Beslay, F. Choteau, D. Crauser, J. M. Becard, E. Plettner, Y. Le Conte, A scientific note on E-β-ocimene, a new volatile primer pheromone that inhibits worker ovary development in honey bees. Apidologie 40, 562–564 (2009).

[25] A. Noël, C. Dumas, E. Rottier, D. Beslay, G. Costagliola, C. Ginies, F. Nicolè, A. Rau, Y. Le Conte, F. Mondet, Detailed chemical analysis of honey bee (*Apis mellifera*) worker brood volatile profile from egg to emergence. PLOS ONE 18, e0282120 (2023). 36809298 10.1371/journal.pone.0282120 PMC9943000

[26] K. S. Traynor, Y. Wang, C. S. Brent, G. V. Amdam, R. E. Page Jr., Young and old honeybee (*Apis mellifera*) larvae differentially prime the developmental maturation of their caregivers. Anim. Behav. 124, 193–202 (2017).

[27] G. Farré-Armengol, I. Filella, J. Llusià, J. Peñuelas, β-Ocimene, a key floral and foliar volatile involved in multiple interactions between plants and other organisms. Molecules 22, 1148 (2017). 28703755 10.3390/molecules22071148 PMC6152128

[28] R. C. da Silva, L. Bestea, G. de Brito Sanchez, M. Giurfa, When the society dictates food search - neural signalling underlying appetitive motivation in honey bees. Curr. Opin. Neurobiol. 89, 102930 (2024). 39490303 10.1016/j.conb.2024.102930

[29] Y. Ji, X. Li, T. Ji, J. Tang, L. Qiu, J. Hu, J. Dong, S. Luo, S. Liu, P. B. Frandsen, X. Zhou, S. H. Parey, L. Li, Q. Niu, X. Zhou, Gene reuse facilitates rapid radiation and independent adaptation to diverse habitats in the Asian honeybee. Sci. Adv. 6, eabd3590 (2020). 33355133 10.1126/sciadv.abd3590 PMC11206470

[30] J. A. Veenstra, L. Rodriguez, R. J. Weaver, Allatotropin, leucokinin and AKH in honey bees and other Hymenoptera. Peptides 35, 122–130 (2012). 22406227 10.1016/j.peptides.2012.02.019

[31] M. J. Marinissen, J. S. Gutkind, G-protein-coupled receptors and signaling networks: Emerging paradigms. Trends Pharmacol. Sci. 22, 368–376 (2001). 11431032 10.1016/s0165-6147(00)01678-3

[32] M. K. C. Ho, Y. Su, W. W. S. Yeung, Y. H. Wong, Regulation of transcription factors by heterotrimeric G proteins. Curr. Mol. Pharmacol. 2, 19–31 (2009). 20021442 10.2174/1874467210902010019

[33] S. Mehra, S. Singh, N. Nagathihalli, Emerging role of CREB in epithelial to mesenchymal plasticity of pancreatic cancer. Front. Oncol. 12, 925687 (2022). 35800049 10.3389/fonc.2022.925687 PMC9253527

[34] A. A. Weger, C. C. Rittschof, The diverse roles of insulin signaling in insect behavior. Front. Insect Sci. 4, 1360320 (2024). 38638680 10.3389/finsc.2024.1360320 PMC11024295

[35] X. Lin, G. Smagghe, Roles of the insulin signaling pathway in insect development and organ growth. Peptides 122, 169923 (2019). 29458057 10.1016/j.peptides.2018.02.001

[36] C. Li, H. Wang, X. Chen, J. Yao, J. Deng, Visual cues and body volatile β-ocimene are used by the blue tiger butterfly *Tirumala limniace* to identify conspecifics during courtship. Behav. Ecol. Sociobiol. 76, 163 (2022).

[37] A. A. Cossé, B. W. Zilkowski, Behavioral responses of Lesser mealworm beetles, *Alphitobius diaperinus*, (Coleoptera: Tenebrionidae) to pheromone components using a wind tunnel dual choice walking bioassay. J. Insect Behav. 28, 202–210 (2015).

[38] S. Schulz, C. Estrada, S. Yildizhan, M. Boppré, L. E. Gilbert, An antiaphrodisiac in *Heliconius melpomene* butterflies. J. Chem. Ecol. 34, 82–93 (2008). 18080165 10.1007/s10886-007-9393-z

[39] A. M. Granero, J. M. G. Sanz, F. J. E. Gonzalez, J. L. M. Vidal, A. Dornhaus, J. Ghani, A. R. Serrano, L. Chittka, Chemical compounds of the foraging recruitment pheromone in bumblebees. Naturwissenschaften 92, 371–374 (2005). 16049691 10.1007/s00114-005-0002-0

[40] M. Molet, L. Chittka, R. J. Stelzer, S. Streit, N. E. Raine, Colony nutritional status modulates worker responses to foraging recruitment pheromone in the bumblebee *Bombus terrestris*. Behav. Ecol. Sociobiol. 62, 1919–1926 (2008).

[41] G. David, S. E. Michael, *Evolution of the insects* (Cambridge Univ. Press, 2005).

[42] R. S. Peters, L. Krogmann, C. Mayer, A. Donath, S. Gunkel, K. Meusemann, A. Kozlov, L. Podsiadlowski, M. Petersen, R. Lanfear, P. A. Diez, J. Heraty, K. M. Kjer, S. Klopfstein, R. Meier, C. Polidori, T. Schmitt, S. Liu, X. Zhou, T. Wappler, J. Rust, B. Misof, O. Niehuis, Evolutionary history of the Hymenoptera. Curr. Biol. 27, 1013–1018 (2017). 28343967 10.1016/j.cub.2017.01.027

[43] S. Cardinal, B. N. Danforth, The antiquity and evolutionary history of social behavior in bees. PLOS ONE 6, e21086 (2011). 21695157 10.1371/journal.pone.0021086 PMC3113908

[44] L. Pecetti, A. Tava, A. Felicioli, M. Pinzauti, E. Piano, Effect of three volatile compounds from lucerne flowers on their attractiveness towards pollinators. Bull. Insectology 55, 21–27 (2002).

[45] N. D. Bryan, L. M. Robert, L. N. John, *The solitary bees: Biology, evolution, conservation* (Princeton Univ. Press, 2019).

[46] A. Maisonnasse, J. C. Lenoir, D. Beslay, D. Crauser, Y. Le Conte, E-β-Ocimene, a volatile brood pheromone involved in social regulation in the honey bee colony (*Apis mellifera*). PLOS ONE 5, e13531 (2010). 21042405 10.1371/journal.pone.0013531 PMC2958837

[47] K. S. Traynor, Y. Le Conte, R. E. Page Jr., Queen and young larval pheromones impact nursing and reproductive physiology of honey bee (*Apis mellifera*) workers. Behav. Ecol. Sociobiol. 68, 2059–2073 (2014). 25395721 10.1007/s00265-014-1811-y PMC4220115

[48] D. R. Nässel, S. F. Wu, Leucokinins: Multifunctional neuropeptides and hormones in insects and other invertebrates. Int. J. Mol. Sci. 22, 1531 (2021). 33546414 10.3390/ijms22041531 PMC7913504

[49] C. Liu, N. Tian, P. Chang, W. Zhang, Mating reconciles fitness and fecundity by switching diet preference in flies. Nat. Commun. 15, 9912 (2024). 39548088 10.1038/s41467-024-54369-w PMC11568147

[50] L. Badisco, P. Van Wielendaele, J. Vanden Broeck, Eat to reproduce: A key role for the insulin signaling pathway in adult insects. Front. Physiol. 4, 202 (2013). 23966944 10.3389/fphys.2013.00202 PMC3735985

[51] Y. Wang, N. S. Mutti, K. E. Ihle, A. Siegel, A. G. Dolezal, O. Kaftanoglu, G. V. Amdam, Down-regulation of honey bee IRS gene biases behavior toward food rich in protein. PLOS Genet. 6, e1000896 (2010). 20369023 10.1371/journal.pgen.1000896 PMC2848551

[52] T. D. Seeley, The honey bee colony as a superorganism. Am. Sci. 77, 546–553 (1989).

[53] R. F. A. Moritz, E. E. Southwick, *Bees as superorganisms: An evolutionary reality* (Springer-Verlag, 1992).

[54] A. L. Toth, S. M. Rehan, Molecular evolution of insect sociality: An eco-evo-devo perspective. Annu. Rev. Entomol. 62, 419–442 (2017). 27912247 10.1146/annurev-ento-031616-035601

[55] S. Kocher, C. Kingwell, The molecular substrates of insect eusociality. Annu. Rev. Genet. 58, 273–295 (2024). 39146360 10.1146/annurev-genet-111523-102510 PMC11588544

[56] A. Dussutour, S. J. Simpson, Communal nutrition in ants. Curr. Biol. 19, 740–744 (2009). 19345104 10.1016/j.cub.2009.03.015

[57] D. G. Le Couteur, S. Solon-Biet, V. C. Cogger, S. J. Mitchell, A. Senior, R. de Cabo, D. Raubenheimer, S. J. Simpson, The impact of low-protein high-carbohydrate diets on aging and lifespan. Cell. Mol. Life Sci. 73, 1237–1252 (2016). 26718486 10.1007/s00018-015-2120-y PMC11108352

[58] Q. Liu, M. Tabuchi, S. Liu, L. Kodama, W. Horiuchi, J. Daniels, L. Chiu, D. Baldoni, M. N. Wu, Branch-specific plasticity of a bifunctional dopamine circuit encodes protein hunger. Science 356, 534–539 (2017). 28473588 10.1126/science.aal3245 PMC5513152

[59] Z. Li, C. Yang, Y. Wu, X. Zhang, X. Zhou, S. Luo, The leucokinin pathway regulates honey bee sugar consumption via *Piezo*. Insect Biochem. Mol. Biol. 186, 104448 (2025). 41232617 10.1016/j.ibmb.2025.104448

[60] Y. Yan, Y. Zhang, Y. Huaxia, X. Wang, P. Yao, X. Guo, B. Xu, Identification and characterisation of a novel 1-Cys thioredoxin peroxidase gene (*AccTpx5*) from *Apis cerana cerana*. Comp. Biochem. Physiol. B Biochem. Mol. Biol. 172-173, 39–48 (2014). 24747012 10.1016/j.cbpb.2014.04.004

[61] T. D. Schmittgen, K. J. Livak, Analyzing real-time PCR data by the comparative CT method. Nat. Protoc. 3, 1101–1108 (2008). 18546601 10.1038/nprot.2008.73

[62] I. Rauluseviciute, R. Riudavets-Puig, R. Blanc-Mathieu, J. A. Castro-Mondragon, K. Ferenc, V. Kumar, R. B. Lemma, J. Lucas, J. Chèneby, D. Baranasic, A. Khan, O. Fornes, S. Gundersen, M. Johansen, E. Hovig, B. Lenhard, A. Sandelin, W. W. Wasserman, F. Parcy, A. Mathelier, JASPAR 2024: 20th Anniversary of the open-access database of transcription factor binding profiles. Nucleic Acids Res. 52, D174–D182 (2024). 37962376 10.1093/nar/gkad1059 PMC10767809

[63] W. Rasband, ImageJ. version 1.54d (U.S. National Institutes of Health, 2011); https://imagej.net/ij/.

[64] S. Chen, Y. Zhou, Y. Chen, J. Gu, Fastp: An ultra-fast all-in-one FASTQ preprocessor. Bioinformatics 34, i884–i890 (2018). 30423086 10.1093/bioinformatics/bty560 PMC6129281

[65] S. Andrews, FastQC: A quality control tool for high throughput sequence data. Bioinformatics 26, 1968–1971 (2010).

[66] D. Kim, B. Langmead, S. L. Salzberg, HISAT: A fast spliced aligner with low memory requirements. Nat. Methods 12, 357–360 (2015). 25751142 10.1038/nmeth.3317 PMC4655817

[67] M. Pertea, G. M. Pertea, C. M. Antonescu, T. C. Chang, J. T. Mendell, S. L. Salzberg, StringTie enables improved reconstruction of a transcriptome from RNA-seq reads. Nat. Biotechnol. 33, 290–295 (2015). 25690850 10.1038/nbt.3122 PMC4643835

[68] M. I. Love, W. Huber, S. Anders, Moderated estimation of fold change and dispersion for RNA-seq data with DESeq2. Genome Biol. 15, 550 (2014). 25516281 10.1186/s13059-014-0550-8 PMC4302049

[69] R. C. Team, *R: A language and environment for statistical computing*. version 3.6.3 (R Foundation for Statistical Computing, 2021); www.R-project.org/.

