# Supplementary material for: Protein craving links larval signals to food provisioning in honey bees
Source: Sci Adv. 2026 May 13;12(20):eaec3855. doi: 10.1126/sciadv.aec3855 (PMC13170622; doi:10.1126/sciadv.aec3855)
Supplement: Supplementary file 1 — Figs. S1 to S6 Legends for tables S1 and S2 Tables S3 to S6 [file sciadv.aec3855_sm.pdf]

Science Advances
AAAS

Supplementary Materials for

Protein craving links larval signals to food provisioning in honey bees

Zhenfang Li *et al.*

Corresponding author: Shiqi Luo, shiqi_luo@cau.edu.cn; Xin Zhou, xinzhou@cau.edu.cn

Sci. Adv. **12**, eaec3855 (2026)
DOI: 10.1126/sciadv.aec3855

This PDF file includes:

Figs. S1 to S6
Legends for tables S1 and S2
Tables S3 to S6

Other Supplementary Material for this manuscript includes the following:

Tables S1 and S2

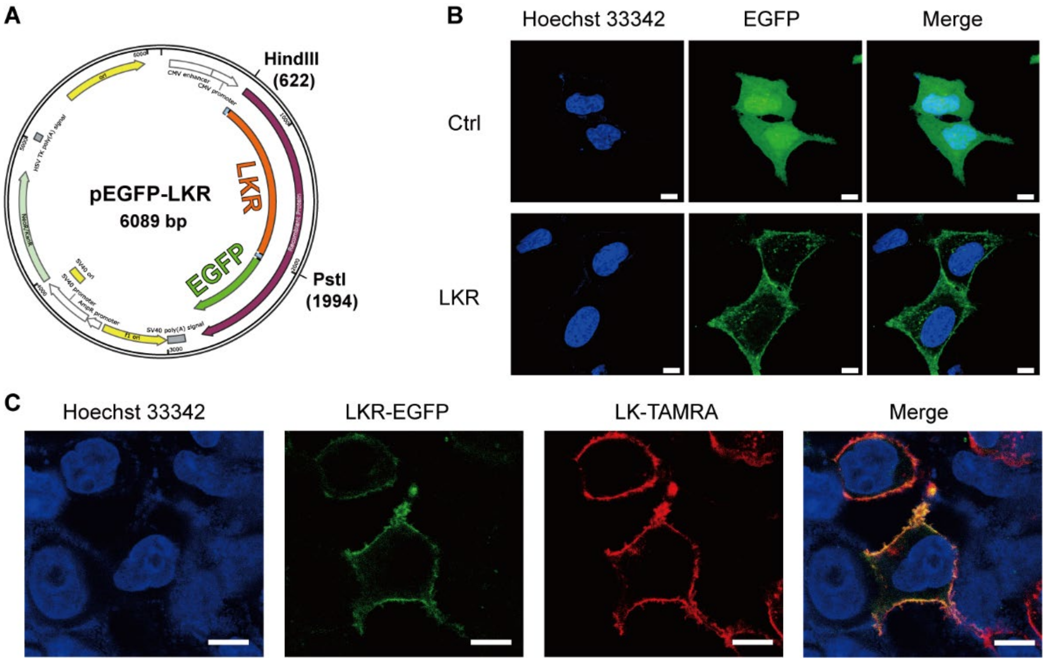

Fig. S1. LK binds to LKR.

(**A**) The pCMV-C-EGFP plasmid containing the LKR protein-coding sequence. **(B)** Expression of the LKR protein on the membrane of HEK293T cells. We transfected the control group with an empty pCMV-C-EGFP plasmid. Scale bar: 10 μm. **(C)** The LK analog bound to LKR on the HEK293T cell membrane. Scale bar: 10 μm.

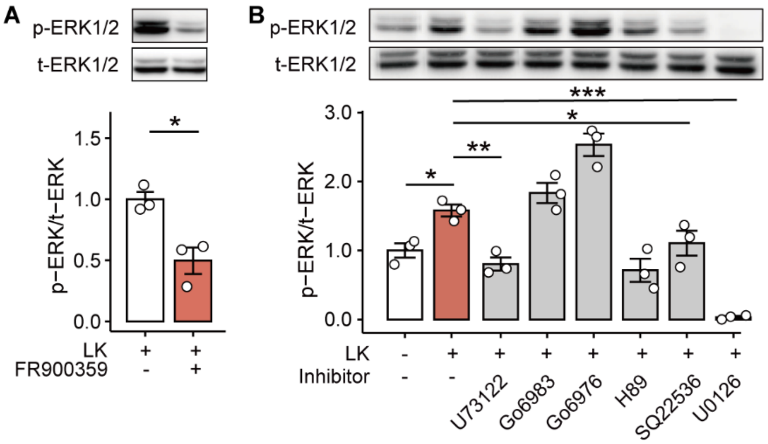

Fig. S2. Binding of LK to LKR activates phosphorylation of ERK1/2.

(A) We found that the Gαq inhibitor FR900359 suppressed LK-induced ERK1/2 phosphorylation. **(B)** We observed that LK activated ERK1/2 phosphorylation, whereas the PLC inhibitor U73122 and the AC inhibitor SQ22536 effectively blocked LK-induced ERK1/2 phosphorylation. We transfected HEK293T cells with pCMV-N-FLAG-LKR plasmid, treated them with LK, and then assessed ERK1/2 phosphorylation (p-ERK1/2) by western blot. We used total ERK1/2 protein (t-ERK1/2) as an internal control. For the control group, we did not apply LK treatment, whereas for

the inhibitor groups, we added inhibitors prior to LK treatment. Data are represented as means ± SEM. N = 3 biological replicates per group. We analyzed data in A and B using a *t*-test. *$P < 0.05$, **$P < 0.01$, and ***$P < 0.001$.

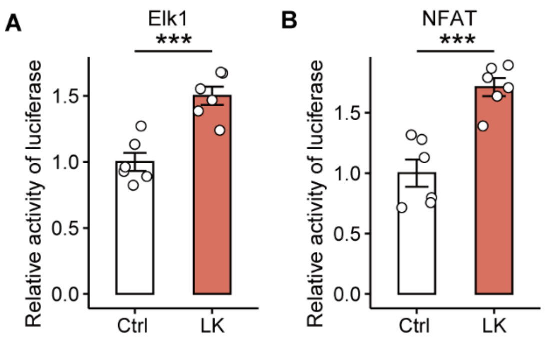

Fig. S3. Activation of downstream transcription factors by the LK signaling pathway.

Activation of Elk1 **(A)** and NFAT **(B)** following LK treatment. We co-transfected HEK293T cells with the pCMV-N-FLAG-LKR plasmid together with either pElk1-TA-Luc or pNFAT-TA-Luc reporter plasmid. We then treated the cells with LK and measured luciferase activity. Data are represented as means ± SEM. N = 6 biological replicates per group. We performed statistical analyses in A and B using a *t*-test. ***$P < 0.001$.

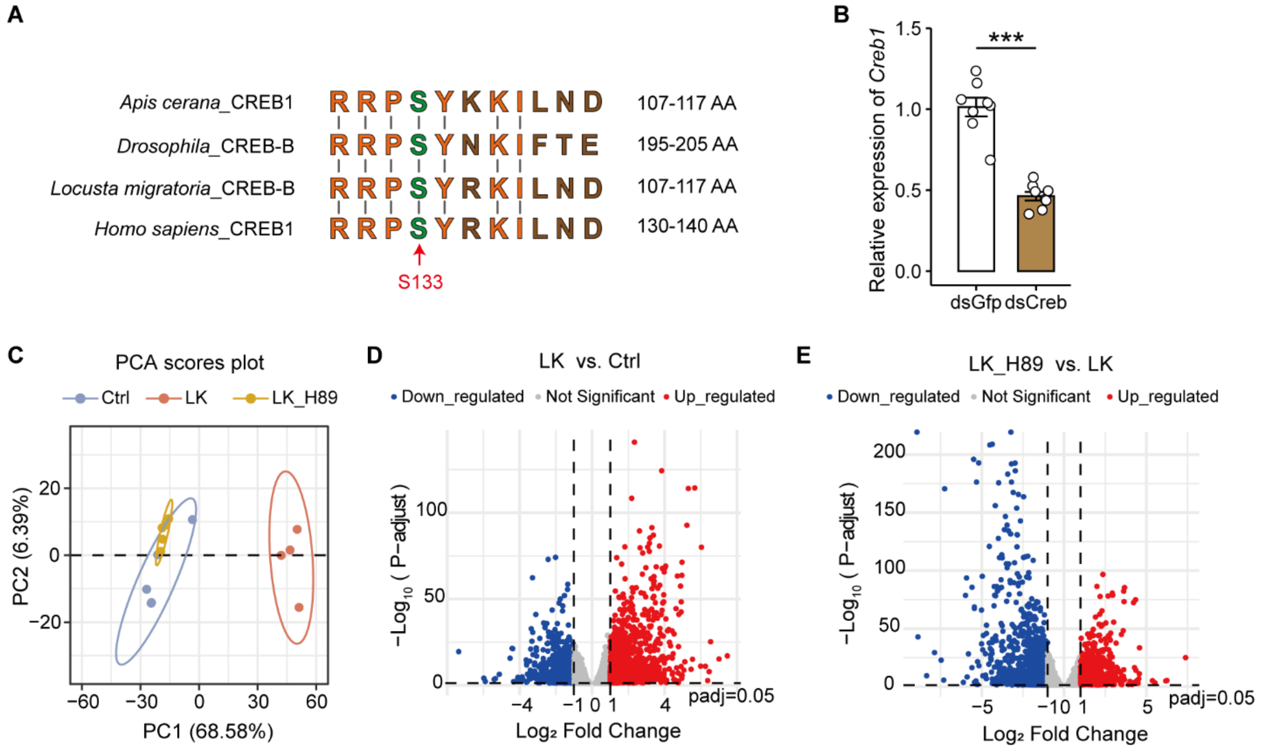

Fig. S4. CREB amino acid alignment, *Creb* RNAi, and head transcriptome profiles of Asian honey bees treated with LK alone or LK plus H89.

(A) Alignment of amino acids surrounding the CREB Ser133 phosphorylation site across different species. **(B)** We quantified *Creb1* expression in bee brains 24 hours after injection of *Creb1* dsRNA, using bees injected with GFP dsRNA as the control. **(C)** PCA clustering of transcriptome data from

bee heads treated with LK and the PKA inhibitor H89. We collected all bee heads 24 hours after injection. (**D-E**) Volcano plots showing the differentially expressed genes (adjusted $P < 0.05$, |fold change| > 2) in bee heads following treatment with LK and H89 for 24 hours. Ctrl: Control group. Each data point in B represents the brain of a single bee. Data are represented as means ± SEM. N = 8 biological replicates per group. We analyzed data in B using a *t*-test. ***$P < 0.001$.

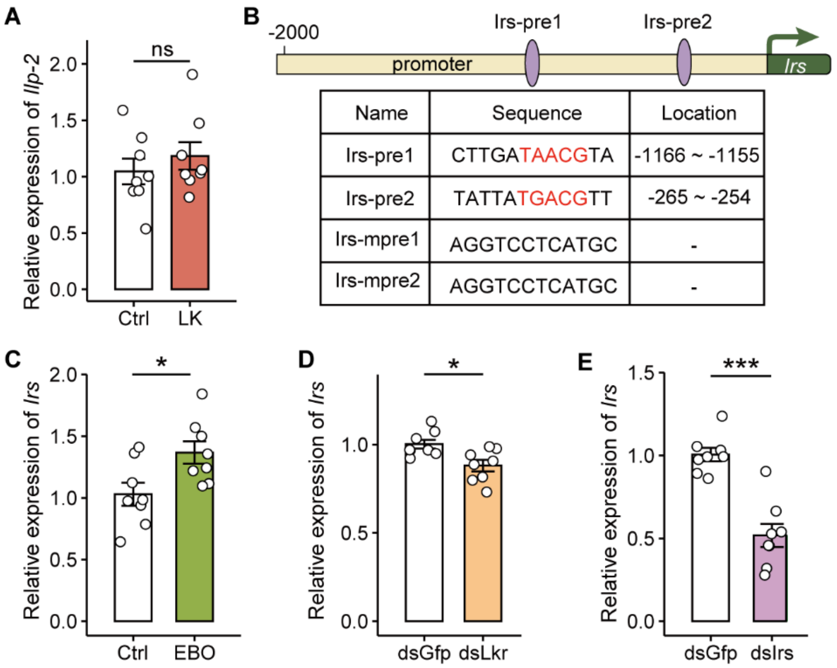

Fig. S5. LK/LKR activates CREB to promote *Irs* transcription in Asian honey bees.

(A) We measured *Ilp2* expression in bee brains 24 hours after LK injection, using bees injected with ddH_2O as the control. **(B)** We predicted two CREB binding sites, Irs-pre1 and Irs-pre2, in the *Irs* promoter region. The CREB motif sequences are highlighted in red. Irs-mpre1 and Irs-mpre2 denote the sequences after mutating the corresponding binding-site regions. (**C-D**) We quantified changes in *Irs* expression in the bee brain 24 hours after EBO treatment **(C)** or *Lkr* RNAi **(D)**. **(E)** We measured *Irs* expression in bee brains 24 hours after *Irs* dsRNA injection, using bees injected with GFP dsRNA as the control. Each data point represents the brain of a single bee. Data are represented as means ± SEM. N = 8 biological replicates per group. We analyzed data in A, C-E using a *t*-test. ns: not significant, *$P < 0.05$, ***$P < 0.001$.

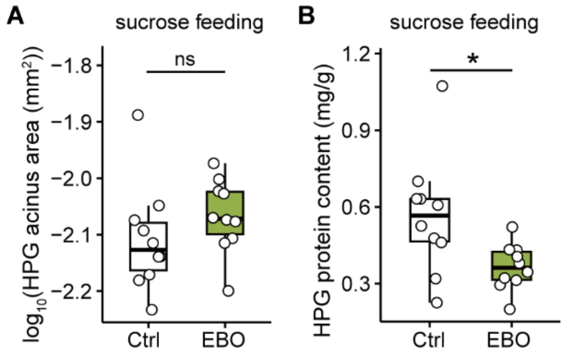

Fig. S6. Effect of EBO on the development of hypopharyngeal glands (HPGs) in adult worker *Apis cerana* bees under pollen-deprived conditions.

(A-B) We found that EBO treatment did not increase the size of acini **(A)** and total protein content **(B)** in the HPGs. Each data point in A and B represents the HPG of a single bee; N = 10 biological replicates per group. We analyzed data in A and B using a *t*-test. ns: not significant, *$P < 0.05$.

Table S1. (separate file)

Summary of differentially expressed genes (DEGs) identified in the heads of bees treated with ddH_2O (control) or LK.

Table S2. (separate file)

Summary of differentially expressed genes (DEGs) identified in the heads of bees treated with LK or co-treated with LK and PKA inhibitor H89.

Table S3. dsRNA primers.

Names	Sequence (5’ – 3’)	Length (bp)	Tm (℃)	Source
dsGfp-F	TAATACGACTCACTATAGGGCGAACGGGTAAACTACCT	383	60	This paper
dsGfp-R	TAATACGACTCACTATAGGGCGATTCTTTTGCTTGTCGGC			
dsLkr-F	TAATACGACTCACTATAGGGTACCTCATTTCATGTGCGCG	413	62	This paper
dsLkr-R	TAATACGACTCACTATAGGGCGCGCGTACACACATGAAAT			
dsCreb-F	TAATACGACTCACTATAGGGACAGCCTAATCAACAATCAGT	447	62	This paper
dsCreb-R	TAATACGACTCACTATAGGGCAGGAACACCTTCGCCTTGG			
dsIrs-F	TAATACGACTCACTATAGGGTGGACAGTTGCTTTAATATTA	292	59	This paper
dsIrs-R	TAATACGACTCACTATAGGGATATATGAAACATTTCGTCTTCA			

Table S4. Primers for RT-qPCR.

Names	Sequence (5'- 3')	Length (bp)	Efficiency	Gene	Source
actin-F	GTTTTCCCATCTATCGTCGG	166	1.01	LOC10799933	Yan et al.(*60*)
actin-R	TTTTCTCCATATCATCCCAG				
q-Lk-F	GTGGCTGCTTCTTTCTATGGTTTCT	97	0.97	LOC107998428	This paper
q-Lk-R	CCCCAGTCCTATTACATCTGCTACA				
q-Lkr-F	AGTTCCGACAGCTGTACTCG	119	0.96	LOC107998395	Ji et al.(29)
q-Lkr-R	CATTCTGCATGCGTCTGGAT				
q-Ilp1-F	TCTTGAAAGATCGGAAGGTTGGA	213	1.03	LOC107997933	This paper
q-Ilp1-R	CACCAAACCGACCAGGACTA				
q-Ilp2-F	ATGGCCAAAAGGGACAGACA	136	1.02	LOC108003129	This paper
q-Ilp2-R	TCATCCATCTCCATTTCTTGGT				
q-sNpf-F	CATTGTTGGCTTCGTTGTTGG	120	0.96	LOC107999041	This paper
q-sNpf-R	AAAGCATTGCGTTGCATCAGA				
q-Npf-F	GACAGATTGATATCTCGACTGGAAA	90	1.04	LOC107993414	This paper
q-Npf-R	TTGGAATACGGTTGCATCGTT				
q-Tk-F	AGGGTGTGCGTGGAAAGAAA	87	0.98	LOC107998479	This paper
q-Tk-R	CCATTGGTGCACGCTTGTTA				
q-Crz-F	TGTTGCATAAAGCTATTAAGATGGT	120	1.10	LOC133667798	This paper
q-Crz-R	TTCCATTTGTCCAGCCATGA				
q-Akh-F	GGAGTTGTCGAATGGGGTGT	109	1.01	LOC108000814	This paper
q-Akh-R	ACATTTTTCGCGCTTCTATCTGT				
q-Creb-F	TAATGCAGCAACAGCAGGTGGA	98	1.03	LOC108001656	This paper
q-Creb-R	CTGCATCTTCTACTACGACACCA				
q-Irs-F	ACTTTGTATTGCGTGGCGAA	98	0.94	LOC108004059	This paper
q-Irs-R	GCTACGTTTCGGTGGTTGTC				
q-InR-1-F	TACTACATGCTCTCCGCCACCAT	83	1.05	LOC108000589	This paper
q-InR-1-R	TTCTCCAGCGTGTCGTTGCAGTAA				
q-InR-2-F	AGCAGACGTTAACCTGGACCAT	98	1.00	LOC108002859	This paper

q-InR-2-R	GGAGGAACGTTCTCTGGACGTT				
q-Mrjp1-F	CGTCCTCTTCTTCGGACTGG	93	1.02	LOC133665627	This paper
q-Mrjp1-R	TGAGCGACGGTACGGATATTG				
q-Mrjp2-F	ACCACCACAGAAAACGGAAGA	104	0.96	LOC107997173	This paper
q-Mrjp2-R	ACGATTAAAGCGTCACCTCTGT				
q-Mrjp3-F	AAAGGCAAAGGTGGTCCTCT	113	0.96	LOC107997171	This paper
q-Mrjp3-R	CTGTCGAATTTGTCGACCGCA				
q-Mrjp4-F	GCAGGACGTGCTAAACAACG	97	0.94	LOC107997172	This paper
q-Mrjp4-R	TGCGCAACGACTATTCCGTA				
q-Mrjp5-F	AATGATGGCCGCAGGAGAAA	110	1.03	LOC107997178	This paper
q-Mrjp5-R	ACAAACTGCGAGAAGCGAGA				

Table S5. Primers for plasmid construction.

Names	Sequence (5' - 3')	Length (bp)	Tm (℃)	Source
LKR-EGFP-F	**GGATCTTCCAGAGAT**AAGCTTATGAAATCCTCCATCATGAATACCT	1404	64	This paper
LKR-EGFP-R	**CTGCCGTTCGACGAT**CTGCAGTAGGCATAATTCTTTTGTATTAAGATCTTG			
LKR-FLAG-F	CCCAAGCTTATGAAATCCTCCATCAT	1379	53	This paper
LKR-FLAG-R	CGGAATTCTAGGCATAATTCTTTTG			
CREB-FLAG-F	CCAAGCTTATGGAAAGTATGGTTGAGGA	826	60	This paper
CREB-FLAG-R	CGGAATTCGTCAGTTTTCGGTTCGCATAGT			
Irspre1-pGL4.10-F	GGGGTACCTTACATATGTATAGTTTCAA	297	55	This paper
Irspre1-pGL4.10-R	CCAAGCTTGCTTTGAACAATACTGT			
Irspre2-pGL4.10-F	GGGGTACCGTCCACCTTGACTTGAAAG	152	60	This paper
Irspre2-pGL4.10-R	CCAAGCTTTATACGCGATATCATGACAA			
mut-Irspre1-pGL4.10-F	TTA*AGGTCCTCATGC*TAGTAATTACTTGATAAATTTTTTACAATAATTAAA	4496	58	This paper
mut-Irspre1-pGL4.10-R	CTA*GCATGAGGACCT*TAATTACTATACTTTTGTCAATACTATTTTCATACA			
mut-Irspre2-pGL4.10-F	CTA*AGGTCCTCATGC*CTATGTAGTGTGAGATATTAAAGAAAGCAGT	4496	64	This paper
mut-Irspre2-pGL4.10-R	TAG*GCATGAGGACCT*TAGAGACGTATGCTTACATGAACACG			

Note: Underline: sites for restriction endonucleases; Bold font: homologous arm; Italic font: mutated sequences.

Table S6. Concentration and duration of inhibitors

Inhibitors	Concentration (μM)	Duration (hours)	Source	Identifier
SQ22536	100	0.5	MedChemExpress	Cat#568500
H89	20	2	Cayman	Cat#10010556
U0126	10	1	Beyotime	Cat#S1901
U73122	10	2	Cayman	Cat#70740
Go6983	10	2	Aladdin	Cat#G129385
Go6976	10	4	Cayman	Cat#13310
FR900359	10	1	Cayman	Cat#33666
